# Repetitive Transcranial Magnetic Stimulation as a Cognitive Rehabilitation Approach for Veterans With Parkinson Disease and Mild Cognitive Impairment: Protocol for a Randomized Sham-Controlled Trial

**DOI:** 10.2196/77241

**Published:** 2026-06-29

**Authors:** Sandra Kletzel, Rama Alsakaji, Alexandria Umbarger, Sadie Walker, Alexandra Aaronson, Amy Herrold, Kalea Colletta, Dulal Bhaumik, Frances Weaver, Theresa Bender Pape

**Affiliations:** 1 Edward Hines Jr VA Hospital Hines, IL United States; 2 Department of Psychiatry, Department of Epidemiology and Biostatistics University of Illinois at Chicago Chicago, IL United States; 3 Edward Hines, Jr VA Hospital Maywood, IL United States; 4 Parkinson School of Health Sciences and Public Health Loyola University Maywood, IL United States; 5 Department of Physical Medicine and Rehabilitation Northwestern University Chicago, IL United States

**Keywords:** Parkinson disease, transcranial magnetic stimulation, neuromodulation, treatment, mild cognitive impairment, military veterans

## Abstract

**Background:**

Parkinson disease (PD) is characterized by motor symptoms as well as progressive cognitive decline leading to long-term functional impairment and diminished quality of life. Mild cognitive impairment in PD (PD-MCI) is a risk factor for developing PD-related dementia. PD-MCI provides a window to assess interventions that can improve cognition. Repetitive transcranial magnetic stimulation (rTMS) shows promise as an effective treatment to improve cognitive performance.

**Objective:**

This study aims to test the safety and feasibility of a 10-session, high-frequency rTMS protocol applied to the left dorsolateral prefrontal cortex and the rTMS efficacy in improving cognitive test performance among veterans with PD-MCI.

**Methods:**

This is a double-blind randomized controlled trial. We will enroll US military veterans with PD-MCI. Participants will be randomized to either active or sham rTMS treatment groups, each with 10 treatment sessions (2 sessions/day). Treatment need not be consecutive; rather, they can be spread across approximately 10 days (eg, Monday, Wednesday, Thursday, Monday, Tuesday, and Wednesday). Participants randomized to active rTMS will receive stimulation applied to the left dorsolateral prefrontal cortex at 110% the resting motor threshold, a 15-Hz rate, 5 seconds per train of pulses, a 10-second intertrial interval, and 40 trains of pulses per session. Each patient will receive approximately 3000 pulses per session. Sham stimulation will be administered at the same parameters as real rTMS; however, no magnetic field will be produced on the placebo side of the active or placebo coil. This protocol was approved by the Edward Hines Jr Veterans Administration Hospital and Jesse Brown Department of Veterans Affairs Medical Center institutional review boards. This study includes a Food and Drug Administration investigational device exemption (G190076).

**Results:**

Safety will be assessed using the number of research-related adverse events experienced by the active rTMS group compared to the sham rTMS group. Feasibility will be assessed using protocol completion rates. To examine preliminary effects of rTMS, participants will complete a standardized neurocognitive battery assessment at baseline, end point, and 1-month follow-up. The primary study outcome is the change in score from baseline to end point on the National Institutes of Health–sponsored Executive Abilities: Measures and Instruments for Neurobehavioral Evaluation and Research Executive Composite score. This project was funded in June 2019, with additional funding secured in April 2024. As of April 2025, a total of 18 veterans with PD-MCI have completed the randomized controlled trial phase. Data collection is ongoing and will be completed by March 2027. We expect the results of this study to be available by March 2028.

**Conclusions:**

The knowledge gained on the safety, feasibility, and efficacy of rTMS will set the stage for future research optimizing therapeutic gains for existing cognitive rehabilitation treatments or developing new and adjunct treatments for PD-MCI.

**Trial Registration:**

ClinicalTrials.gov NCT03836950; https://clinicaltrials.gov/study/NCT03836950

**International Registered Report Identifier (IRRID):**

DERR1-10.2196/77241

## Introduction

### Background and Rationale

Parkinson disease (PD) is a progressive, degenerative neurological and neuropsychiatric disorder affecting over 4 million people worldwide over the age of 50 years [1,2]. PD management involves treating motor and nonmotor (eg, cognitive and mental health) symptoms. Longitudinal studies indicate that 20% to 55% of patients with PD currently have mild cognitive impairment (PD-MCI, mild cognitive impairment in PD; ie, mild neurocognitive disorder due to PD) [3]; however, there is a paucity of effective treatments addressing this cognitive decline.

Executive function impairment is a hallmark symptom of PD and is typically associated with diminished quality of life, reduced health status, hindrance of everyday function, and increased caregiver burden [4,5]. On objective cognitive assessments, people living with PD exhibit impairments in aspects of executive function, such as trial and error learning, planning, response monitoring, and set shifting [6]. Other cognitive domains that can be affected by PD include attention, working memory, language, memory, and visuospatial function [7]. However, executive dysfunction is the most prominent cognitive concern, and deficits in this area may underlie impairments in other cognitive domains [6]. Unfortunately, while effective treatments exist for motor symptoms of PD, there are limited treatment options for PD-related cognitive dysfunction.

Repetitive transcranial magnetic stimulation (rTMS), a nonpharmacological and noninvasive cortical stimulation technique that can be used to induce neuroplasticity in targeted brain regions and networks, may serve as a possible treatment option for PD-related cognitive dysfunction [8-10].

To target cognitive networks using rTMS, cortical site/sites for stimulation must be chosen.

PD is associated with dysfunction of the frontal-striatal pathways, which compromises frontal cortical regions, including the dorsolateral prefrontal cortex (DLPFC). Neuroimaging studies report that higher executive function scores are associated with stronger connectivity related to the DLPFC among patients with PD [11]. Mild executive dysfunction in PD is also associated with abnormal suppression of the DLPFC at rest [11]. On the basis of this evidence, high-frequency stimulation to the DLPFC is a logical approach for a high-frequency rTMS protocol. Indeed, reviews of the literature indicate that high-frequency rTMS over the DLPFC for multiple sessions can improve cognitive function. A review by Jiang et al [9] concluded that high-frequency rTMS over the DLPFC for multiple sessions (compared to a single session) had a significant effect on executive function in patients with PD.

Initial rTMS studies that targeted the DLPFC and measured change in cognitive performance used a single-session protocol in patients with PD with intact cognitive function (PD-N). One session of high-frequency (25 Hz) rTMS to the right DLPFC among 10 patients with PD-N who were temporarily taken off their PD motor medications showed improvements in executive function (ie, strategic planning), measured using the Tower of London test [12]. The same research group found that 1 session of high-frequency (10 Hz) rTMS to the left DLPFC in another 10 participants with PD-N off their PD medications did not improve executive function, working memory, or psychomotor speed [13].

Two initial randomized double-blind placebo-controlled trials examined the effects of multisession rTMS targeted to the DLPFC on depression (primary outcome) and cognitive function (secondary outcome) among patients with PD-N. Both randomized controlled trials (RCTs) applied high-frequency (5 Hz [14] or 15 Hz [15]) rTMS to the left DLPFC over the course of 10 sessions. Both studies reported improved depression and cognitive function, particularly executive function as measured using the Stroop test [14,15] and Wisconsin card sorting test [15]. These cognitive improvements were maintained for 20 [15] to 30 [14] days after the last rTMS session.

Three initial RCTs examined the effects of multisession rTMS on cognitive function (primary outcome) among patients with PD-MCI. Buard et al [16] reported no between-group differences on the total score on the Dementia Rating Scale–2 in a study that administered high-frequency (20 Hz) bilateral DLPFC rTMS at 90% the resting motor threshold (RMT) for 10 sessions over 2 weeks. In contrast, 2 studies reported the effects of 10 sessions of high-frequency intermittent theta-burst (a patterned form of rTMS) targeted to the left DLPFC. Trung et al [17] reported improved visuospatial function after a 30-day follow-up in the active group vs a sham group. He et al [18] reported improvements in global cognitive function and in the Repeatable Battery for the Assessment of Neuropsychological Status domains of immediate and delayed memory and visuospatial ability. The improvements persisted for at least 3 months after the stimulation [18]. This evidence supports our selection of 10 sessions of rTMS to improve cognitive function.

The selection of the DLPFC as the site for stimulation is supported further by findings from a systematic review conducted by Guse et al [8]. This review focused on the effectiveness of using high-frequency rTMS targeting the DLPFC to enhance cognition in populations with acute neurological and neurodegenerative conditions and healthy populations. On the basis of their review of changes in cognitive test performance, the authors concluded that rTMS at 10, 15, or 20 Hz applied to the left DLPFC within a range of 10 to 15 successive sessions and a motor threshold of 80% to 110% is most likely to cause significant improvement in cognitive performance, including executive function. Moreover, they found that patients had better improvements than healthy controls. Many of these aforementioned studies targeted the DLPFC, and most used 10 to 20 Hz of rTMS with at least 5 sessions.

There is considerable evidence that rTMS performed in compliance with the recommended safety guidelines [19-21] is reasonably safe with mild side effects. The most serious risk of rTMS is seizures; however, the risk of seizure in healthy persons is exceedingly low [22]. A safety review indicates no reported rTMS-induced seizures in the PD population and that the more common safety issues related to rTMS in PD are similar to those in the non-PD population [23]. The most common side effects include a mild transient headache, scalp pain at the stimulation site, neck pain, tooth pain, transient changes in audition, and syncope. While rTMS is considered a safe procedure when provided within safety guidelines, it is important to remember that it is an experimental treatment in PD without Food and Drug Administration (FDA) clearance. Thus, there may be unknown risks. Safety is of the highest priority; thus, it is critical that investigators measure and report on the presence or absence of adverse events [19]—this will help address existing gaps in the PD rTMS safety literature.

### Objectives

The research objective is to examine the therapeutic effects of rTMS on cognitive function in veterans with PD-MCI. The central hypothesis is that rTMS will safely improve cognitive function in PD-MCI.

There are 2 aims of the study. Aim 1 is to determine the safety and feasibility of rTMS treatment. The hypothesis is that 10 high-frequency rTMS sessions will be (1) safe, as evidenced by the lack of difference in the number of research-related adverse events experienced by the active rTMS group compared to the sham rTMS group; and (2) feasible, as indicated by participant protocol completion rates.

Aim 2 is to determine the behavioral effects of rTMS treatment. A battery of neuropsychological tests will be administered at baseline, end point (after the 10th rTMS session), and the 1-month follow-up. The hypothesis is that active rTMS, compared to sham rTMS, will enhance cognitive test performance after rTMS completion and at the 1-month follow-up. The primary outcome will be change in executive function (ie, end point minus baseline) as measured using the National Institutes of Health–sponsored Executive Abilities: Measures and Instruments for Neurobehavioral Evaluation and Research (NIH-EXAMINER) Executive Composite score.

To address the study objectives, a prospective parallel-group RCT will be conducted. A convenience sample of veterans (N=66) who have been screened and diagnosed with PD-MCI will be randomly assigned to 1 of 2 groups: active rTMS or sham rTMS to the left DLPFC.

## Methods

### Participants, Interventions, and Outcomes

#### Study Setting

Participants will be recruited from Chicago metropolitan area US Department of Veterans Affairs (VA) hospitals: Edward Hines Jr. Veterans Administration Hospital (hereafter referred to as “Hines VA”) and Jesse Brown VA Medical Center in the state of Illinois, United States. The intervention will be conducted at the Hines VA.

#### Eligibility Criteria

Veteran participants will be included if they have a diagnosis of PD (idiopathic or atypical) as determined by a VA neurologist and are aged 50 years or older. They must be on stable medication (ie, no changes in medication and/or dose in the previous month) and are expected to remain on stable medication for the duration of the study. Participants must demonstrate decision-making capacity prior to enrolling in the study, that is, verbally demonstrate (1) understanding of the purpose and activities required in the study, (2) appreciation for the potential risks and benefits, (3) appreciation of alternative choices other than participating in the study, and (4) expression of choice about whether they want to participate. PD-MCI will be determined using the International Parkinson and Movement Disorder Society level 2 diagnostic criteria [24], as detailed in Table 1. Patients will be excluded if they exhibit dementia (score of <21 on the Montreal Cognitive Assessment [MoCA]) or severe depression (score of >29 on the Beck Depression Inventory–II). Patients will be excluded if they have a history of deep brain stimulation surgery. Other reasons for exclusion are based on contraindication for magnetic resonance imaging (MRI), including implanted cardiac pacemaker or defibrillator, implanted medical pump, increased intracranial pressure, metal in the eyes or face, or shrapnel or bullet remnants in the brain. Patients who have contraindications for rTMS, including history of psychotic spectrum disorders such as bipolar and schizophrenia, history of suicide attempts, metal or medical implants in the head or neck, increased intracranial pressure, or resting head tremor, or are taking medications that lower seizure threshold will also be excluded. Medication lists and the current evidence in the literature for safety profiles of medications will be reviewed by the principal investigator (PI) and study team clinicians to determine eligibility

**Table 1:** Defining mild cognitive impairment in Parkinson disease (PD-MCI)^a^.

Assessment name	Level	Construct measure	Reliability
Montreal Cognitive Assessment	1	Global cognition	Cronbach α=0.83 [25]
Stroop color word test	2	Attention and working memory	Test-retest reliability=0.73 [26]
Letter-number sequencing	2	Attention and working memory	Test-retest reliability=0.79 [27]
Wisconsin card sorting test	2	Executive function	Cronbach α=0.83 [28]
Trail making test	2	Executive function	Test-retest reliability=0.76-0.94 [29]
Hopkins Verbal Learning Test–Revised	2	Memory	Test-retest reliability=0.74 [30]
Brief Visuospatial Memory Test–Revised	2	Memory	Test-retest reliability=0.97-0.98 [31]
Judgment of Line Orientation test (short version)	2	Visuospatial function	Cronbach α=0.75-0.77 [32]
Hooper Visual Organization Test	2	Visuospatial function	Cronbach α=0.88 [33]
Similarities test	2	Language	Cronbach α=0.85 [34]
Boston Naming Test short form 4	2	Language	Cronbach α=0.67 [35]

^a^International Parkinson and Movement Disorder Society level 2 diagnostic criteria [24] allow for the diagnosis and subtyping of PD-MCI using a full battery of neuropsychological tests, which include at least 2 tests in each of the 5 domains most affected in Parkinson disease. For this study, we will define impairment as 1 SD below the normative mean in 2 tests in the same domain or any 2 tests in different domains.

#### Intervention

Participants will receive a total of 10 active or sham rTMS sessions. They will be scheduled to receive 2 sessions per day. Treatment days do not need to be consecutive; rather, they can be spread across approximately 10 days (eg, Monday, Wednesday, Thursday, Tuesday, and Wednesday). Participants randomized to active rTMS will receive stimulation applied to the left DLPFC at 110% the RMT and a 15-Hz rate, 5 seconds per train, 10-second intertrial interval, and a total of 40 trains per session. Each patient will receive approximately 3000 pulses per session. Sham stimulation will be administered at the same parameters as the real rTMS; however, no magnetic field will be produced on the placebo side of the active or placebo coil. Patients will be blinded as to which group they are in (treatment or sham). Participants will be unblinded to their treatment group after the last participant completes the study via a letter that will be mailed to the veterans (see details in the rTMS Sessions section).

Each participant will be instructed to take their PD medication within an hour before their first scheduled session of the day. rTMS will be administered at approximately the same time each day, with an hour break in between sessions.

#### Preparation for rTMS Session

##### Neuroimaging for Neuronavigation

Participants will undergo a structural MRI scan to locate the left motor cortex and left DLPFC using the Localite TMS Navigator neuronavigation software. The MRI sequence will include a high-resolution sagittal 3D T1-weighted volume acquired on a 3-T Siemens Skyra scanner. This scan will take approximately 8 minutes and will be acquired using the following parameters: repetition time of 2300 ms, echo time of 2.91 ms, flip angle of 9°, field of view of 256 mm, time inversion of 900 ms, and voxel size of 1 mm^3^.

##### Determining the Intensity of the rTMS Stimulation

To determine RMT, the participant’s T1 structural brain image will be loaded onto the Localite TMS Navigator system. Single-pulse transcranial magnetic stimulation (TMS) using the MagVenture C-B60 coil will be applied to determine the optimal scalp location (“hot spot”) to activate the abductor pollicis brevis on the right hand to determine the RMT. The RMT will be the lowest stimulus intensity necessary to produce a motor-evoked potential of peak-to-peak amplitude of 50 μV or higher in 5 of 10 subsequent trials. Motor-evoked potential will be recorded using surface electrodes.

##### Localization of the DLPFC

The target for stimulation to the left DLPFC will be measured as approximately 5.5 cm anterior from the individual’s hot spot in the left motor cortex, as determined during motor thresholding. The DLPFC target will be premarked and loaded onto the Localite TMS Navigator system at the beginning of every treatment session to ensure reproducibility and consistency at the stimulation site.

##### rTMS Sessions

A MagVenture MagProX100 with MagOption stimulator and MagPro Cool-B65 active or placebo coil will be used to administer active and sham rTMS. To maintain blinding, the active or placebo coil will be connected to a placebo noise generator. This generator produces the same pattern of clicking sounds produced when the active stimulation is administered. For every treatment session, the participant and treatment providers will wear headphones connected to the placebo noise generator so that everyone in the treatment room hears the same noise regardless of whether the treatment is active or placebo. To simulate the somatic sensations of active TMS, surface electrodes will be placed on the left side of the forehead of every participant; an electrical sensation will be produced that coincides with the delivery of the pulses (active or sham) in the stimulation protocol.

#### Outcomes

The primary study outcome is the change in score from baseline to end point on the NIH-EXAMINER Executive Composite score. The composite score is advantageous because it combines performance across various aspects of executive function consistently reported to be impacted by PD [36]. It is derived using item response theory, which can improve sensitivity and increase statistical power over standard scoring methods [37]. Seven tests in the NIH-EXAMINER will be used to compute the composite score, as detailed in Table 2. This battery takes approximately 30 minutes to administer, and each test has 3 alternate forms to help avoid practice or learning effects when administered multiple times during the study.

**Table 2:** Study assessments^a^.

Assessment name		Construct measure
**Primary outcome**		
	NIH-EXAMINER^b^ Executive Composite score	Executive functioning [38]
**Secondary outcomes**		
	Dot counting^c^	Working memory
	1-back task^c^	Working memory
	2-back task^c^	Working memory
	Flanker task^c^	Cognitive control
	Set shifting^c^	Cognitive control
	Phonemic Fluency and Category Fluency^c^	Verbal fluency
	Category^c^	Verbal fluency
	HVLT-R^d^	Verbal memory [30]
	HVOT^e^	Visuospatial function [33]
	SDMT^f^	Processing speed [39]
	PD-CFRS^g^	Real-life function [40]
	AM-PAC^h^ applied cognitive domain questionnaire	Real-life function [41]
	BDI-II^i^	Depression [42]
	BAI^j^	Anxiety [43]
	AES^k^	Apathy [44]
	MoCA^l^	Global cognition [25]

^a^The estimated time to complete the study assessments is 2.5 hours (30 minutes for the National Institutes of Health–sponsored Executive Abilities: Measures and Instruments for Neurobehavioral Evaluation and Research and 2.5 for all the study assessments).

^b^NIH-EXAMINER: National Institutes of Health–sponsored Executive Abilities: Measures and Instruments for Neurobehavioral Evaluation and Research.

^c^Seven NIH-EXAMINER subtests that contribute to the Executive Composite score that will also be assessed as secondary outcomes.

^d^HVLT-R: Hopkins Verbal Learning Test–Revised.

^e^HVOT: Hooper Visual Organization Test.

^f^SDMT: Symbol Digit Modalities Test.

^g^PD-CFRS: Parkinson Disease Cognitive Functional Rating Scale.

^h^AM-PAC: Activity Measure for Post-Acute Care.

^i^BDI-II: Beck Depression Inventory–II.

^j^BAI: Beck Anxiety Inventory.

^k^AES: Apathy Evaluation Scale.

^l^MoCA: Montreal Cognitive Assessment.

The NIH-EXAMINER establishes a 3-factor model defined by cognitive control, working memory, and fluency. A confirmatory factor analysis indicates that these 3 factors load on to 1 factor: the Executive Composite score [38]. The ecological validity of the composite score was assessed in 225 patients with various neurological conditions, including PD [45]. Exhibiting concurrent validity, the composite score is a robust predictor of real-world executive behavior, as measured using the Frontal Systems Behavior Scale [36]. Furthermore, poor composite scores correlate with atrophy in frontal cortical regions that mediate executive function [36]. In patients with PD, the sensitivity of the NIH-EXAMINER was compared to that of other commonly used tests of executive function recommended by the International Parkinson and Movement Disorder Society task force on PD-MCI [24]; the NIH-EXAMINER Executive Composite score was the most sensitive and specific in detecting cognitive impairment, with an effect size of 0.94 [36].

Secondary outcomes include change from baseline to end point in the cognitive control composite score from the NIH-EXAMINER, working memory composite score from the NIH-EXAMINER, delayed verbal memory score for the Hopkins Verbal Learning Test–Revised [30], total score on the oral version of the Symbol Digit Modalities Test [46], total score on the Hooper Visual Organization Test [33], total score on the Beck Depression Inventory–II [47], total score on the Beck Anxiety Inventory [48], total score on the Apathy Evaluation Scale [49], total score on the MoCA [50], total score on the PD Cognitive Functional Rating Scale [40], and total score on the Activity Measure for Post-Acute Care applied cognitive domain questionnaire [41].

#### Participant Timeline

**Figure 1:**
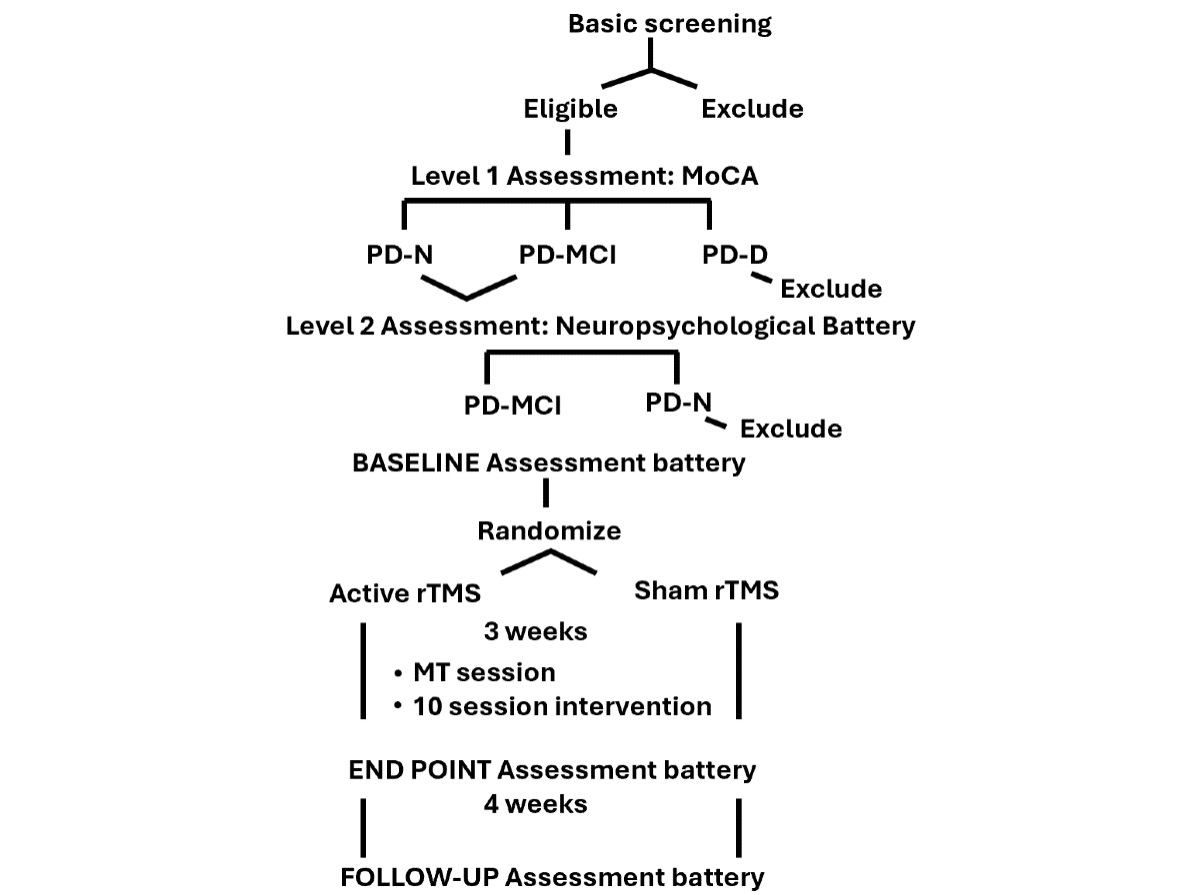
Study flow diagram. Recruitment will start with basic screening of medical records and recruitment phone calls. Those who are eligible and consent to the study will undergo a level 1 cognitive assessment using the Montreal Cognitive Assessment (MoCA) to screen for possible mild cognitive impairment in Parkinson disease (PD-MCI). On the basis of the MoCA total scores, 26 or higher indicates Parkinson disease with intact cognitive function (PD-N), 21 to 25 indicates PD-MCI, and less than 21 indicates Parkinson disease–related dementia (PD-D) [25,44]. Those scoring 21 or higher on the MoCA will then undergo a level 2 cognitive assessment using a full neuropsychological battery to determine PD-MCI diagnosis (see Table 1 for details). Eligible participants will complete the baseline assessment battery of tests and will then be randomized to active or sham repetitive transcranial magnetic stimulation (rTMS). Over approximately 2 weeks, the participants will complete the motor threshold (MT) session to determine the dose of rTMS and target dorsolateral prefrontal cortex treatment site, and 10 sessions of rTMS will be completed. Within approximately 1 week of completing the last session of rTMS, participants will complete end-point assessments. Approximately 4 weeks later, the participants will complete the follow-up assessments.

Time schedule of enrollment, interventions (including any run-ins and washouts), assessments, and visits for participants. A schematic diagram of the study timeline is shown in Figure 1.

#### Sample Size Determination

Effect sizes for rTMS on cognition in the PD literature are variable, with a range of 0.04 [51] to 0.36 [14]. However, there are several limitations to these studies that lead us to believe that the effect size will be larger in the population with PD-MCI. First, given that the existing literature has studied patients with PD-N, it is reasonable to expect that cognitive effects in these studies may have been limited by a ceiling effect such that cognitive function could not have improved any further [8]. In addition, not all the existing PD studies have used neuronavigation, so variability was likely increased in terms of the actual site being stimulated [52]. Such treatment variability can increase variability in cognitive outcome measures and, thus, lower the effect size [53]. Finally, all existing PD literature examining cognitive effects has used 100% the RMT or less. As people age, cortical atrophy occurs, leading to increased scalp-to-cortex distance. This atrophy occurs more in the prefrontal regions compared to the motor cortex [54]. If this is not compensated for, it can weaken rTMS stimulation intensity. Thus, effects observed using relatively lower stimulation intensity (80%-100%) likely underestimate the benefits of rTMS for executive function in PD studies. One way to compensate for this is to increase the RMT to above 100%.

The limitations of existing data indicate that rTMS effect sizes on cognition in Alzheimer disease are likely more accurate for sample size determination in this study. Similar to PD, patients with Alzheimer disease experience cognitive deficits and have overlapping brain regions undergoing neurodegeneration. A meta-analysis [55] estimated a moderate effect size of 0.48 in terms of the benefits of rTMS (with many of the included studies targeting the DLPFC) for cognition among older patients with cognitive impairment related to Alzheimer disease.

Taking all this into consideration, as well as the fact that this is a small-scale trial, the effect size of 0.48 found in the aforementioned meta-analysis [55] was estimated for this RCT. The sample size was determined using a 2-level mixed-effects longitudinal model with random participant intercept and random participant slope. The end point effect size was fixed at 0.48, which is considered moderate. The participant intercept and slope variances were fixed at 2.3 and 0.3, respectively. In addition, the error variance was fixed at 2.0, which should be considered moderate. Using this combination of parameters, we determined that a sample size of 71 per group (active and sham) considering an attrition rate of 10% will be needed to achieve 80% power while maintaining the type 1 error rate at 5%. The RMASS software was used for this power analysis [56]. Hence, a total of 156 participants will be recruited for this proposed study.

#### Recruitment

Military veteran participants will be recruited through VA national databases, clinician referrals, and flyers placed in the 2 participating VA hospitals.

### Assignment of Interventions

Participants will be randomized to either the active or sham rTMS. The sequence will be produced by the study biostatistician using an online random number generator. There will be no stratification. An unblinded study team member will assign the participant number series codes from the MagVenture study master edition. These participant codes correspond to randomized treatment numbers that are entered into the MagVenture MagProX100 stimulator before each treatment session.

Participants and study team members administering and scoring outcome measures, as well as the study biostatistician, will be blinded to group assignment. Researchers blinded to treatment group will administer outcome measurements. The effectiveness of blinding will be evaluated using data collected from end point questionnaires that assess perception of the intervention.

Unblinding will occur after participants have completed the study.

### Data Collection, Management, and Analysis

#### Data Collection Methods

Figure 1 [25,50] illustrates plans for assessment and collection of outcomes during screening, baseline, end point, and follow-up visits.

#### Data Management Methods: Statistical Methods

For safety outcomes, the Poisson distribution for the number of research-related adverse events will be fitted for each treatment group. Negative binomial distributions will be used in case overdispersion is present. The mean parameters (λ) of these 2 distributions will be tested to see whether any difference in the average number of adverse events in each treatment group exists. Lack of significance will suggest that active rTMS is as safe as sham rTMS in terms of adverse events. To further compare the trend in adverse events between the 2 groups, we will use a 3-level mixed-effects Poisson regression model, and the group-by-time interaction parameter will be tested to examine whether active rTMS has more adverse events than sham rTMS over time.

For neuropsychological outcomes, all data will be analyzed using a 2-level longitudinal mixed-effects model with random participant effects to incorporate correlation of measurements nested within the same participant. The model will also include covariates such as medication dose and anxiety and depression levels. Parameters will be estimated using measurements from all 3 time points (ie, baseline, end point, and follow-up). The group-by-time interaction parameter will be used to examine the difference in the trends of the active rTMS group compared to the sham rTMS group. The significance of this parameter will indicate whether treatment is efficacious in terms of executive function over time when adjusted for the identified covariates. To assess the immediate effects of rTMS, one contrast using means of test scores at baseline and end point from the experimental group will be obtained, and the other contrast will be obtained from those same types of means for the control group. These 2 contrasts will then be tested using a 2-tailed *t* test to compare the change scores of the 2 groups. To assess sustainability of the rTMS effects, contrasts using means of test scores at end point and follow-up will be obtained for each group, and the contrast will then be compared using a *t* test. Estimates of variance components from mixed-model analysis will be used to calculate test statistics. To account for the many-to-many comparisons of secondary outcomes, we will use a multivariate mixed-effects model with the aforementioned covariates. For every secondary outcome component, there will be fixed as well as random components. The random component will address the within-subject correlation and the heterogeneity between multiple outcomes. Every component will have its own trend, which means that the recovery curve may be outcome specific. The group-by-time interaction parameter of each secondary outcome will be tested to examine whether cognitive function in the active rTMS group is different from that in the sham rTMS group. The false discovery rate will be addressed using Benjamini and Hochberg [57] procedures, with a false discovery rate level of 0.05. Data analysis will be performed using the SAS PROC MIXED statement (version 9.3; SAS Institute). This modeling process will be reported for each outcome of interest for the safety and neuropsychological assessments. Every effort will be made to avoid missing data; we will try to understand the missingness pattern and why and how the missingness occurred. No missing value imputation technique will be used if Little test supports that data are missing completely at random. Otherwise, proper missing data imputation techniques (eg, imputation with mean, nearest neighbor, or last observation carried forward) will be used.

Potential moderators of the effects of rTMS will be explored through Pearson correlations and regression analyses. Factors include, for example, age at study enrollment, age at PD onset, disease duration, MoCA score, levodopa equivalent dose, and change in mood (eg, depression, anxiety, and apathy). Blinding success will be reported alongside the primary results.

### Monitoring

#### Data Monitoring

There is no data monitoring committee for this pilot study. There is a medical monitor with whom the study team consults for safety.

#### Harms: Safety Monitoring

All study procedures and parameters are in accordance with the current safety and application guidelines for rTMS [19,21]. Treatment will be delivered by personnel trained in the administration of rTMS. An rTMS data safety monitoring sheet (DSMS), adverse event tracking log, medication log, and sleep log for each participant will be kept to monitor session attendance and any side effects or adverse events according to prespecified criteria. The Columbia-Suicide Severity Rating Scale will also be administered at the baseline, end point, and follow-up assessments.

The DSMS contains a customized severity indicator scale with before-and-after variables, including rates of change from baseline in vital signs (temperature, blood pressure, heart rate, and oxygen saturation levels), fatigue, tinnitus, sleep, dizziness, nausea, vomiting, confusion, seizure, syncope (fainting), headache, neck pain, skin integrity of the scalp, and movement. PD-related motor assessments, including speech, facial expression, finger taps, rapid alternating movement of the hands, tremor at rest, leg agility, ability to rise from a chair, gait, and body bradykinesia and hypokinesia, will also be conducted before and after each rTMS session and compared to baseline.

An adverse event tracking log will also be developed for this project for further safety monitoring. All serious and nonserious adverse events will be tracked on this log.

Sleep disruption and deprivation is associated with a lower seizure threshold, which may put the participant at a heightened seizure risk when receiving rTMS. Therefore, the sleep log asks the participants to record total hours of sleep at night and total minutes of nap time during the day. After the baseline assessment, a study team member will provide a sleep packet (collection of blank sleep logs) for the participants. The participants will then receive instructions on how to use the log. Data collected before the first rTMS session will be averaged, and variance will be documented and used as the baseline for the DSMS. At least 3 days of “total hours of sleep” will be required for the participant to complete the first session of rTMS. During the study period, self-reported sleep based on the sleep log will be checked before the intervention is administered for a given day.

Change in medication can also affect the seizure threshold. Therefore, a personalized medication log is generated for each participant. A thorough review of active medications (including prescriptions, over-the-counter medications, and supplements) will be conducted by a study team member, checked against the medical record, and reconciled with the participant to generate an accurate log of medications the participant is taking. After the baseline assessment visit, a study team member will provide a packet (collection of blank medication logs) for the participants. The participants will then receive instructions on how to use the log. During the study period, self-reported medication intake based on the medication log will be checked before the intervention is administered for a given day.

The PI has the right to terminate participation if a participant becomes uncooperative or unwilling to complete study tests, if the participant is experiencing undue stress from study procedures, or if the participant has medical problems that interfere with completion of study tests. As the long-term effects of the experimental treatment are unknown, if a participant withdraws before study completion, we recommend that they complete follow-up assessments.

### Ethical Considerations

#### Human Subject Ethics Approval

This study was approved by both the Hines VA (1337782) and Jesse Brown VA Medical Center (1395002) institutional review boards (IRBs) and is registered on ClinicalTrials.gov (NCT03836950). This study has also received an FDA investigational device exemption (G190076). Important modifications to the protocol will be communicated to the sponsor, IRB, and FDA for approval before implementation. The registration on ClinicalTrials.gov will also be updated.

#### Informed Consent

Consent will be obtained by trained study team members. All study participants will provide informed consent before completing research procedures. Participants will be given the option to opt out of consenting or research procedures at any time without loss or benefits. Research participants will have access to research staff to assist with any questions or concerns until understanding is achieved to the judgment of the individual asking the questions. A copy of the signed consent form will be provided to the participants. The consent form will indicate that data will be placed in a Hines VA IRB-approved data repository for potential future use. Only IRB-approved studies with approved data use agreements can request and use the research data for ancillary studies.

#### Privacy and Confidentiality

All hard copies of assessments will include a unique participant identification number and the date the assessment was completed. Thus, data will be deidentified. The only identifiable information will be the demographic questionnaire and contact information. The demographic questionnaire will be kept in a locked filing cabinet in the study PI’s locked office. The participants’ names and contact information will be linked to their unique study identification number and saved electronically on a secure VA crosswalk server at the Hines VA. This secure crosswalk file is the only place where the participants’ names will be linked with their unique participant identification number. The study PI will have control over the research team members who are allowed access to the crosswalk file.

#### Compensation Details

Participants will receive up to US $230 for completing all study procedures. They will receive US $25 after completing the screening visit, US $25 after completing baseline assessments, US $30 for completing the MRI scan, US $50 after the 1st rTMS session, US $50 after the 10th rTMS session, US $25 after completing end-point assessments, and US $25 after completing follow-up assessments. This compensation is intended to facilitate participation without adding undue influence.

## Results

This project was funded in June 2019, with additional funding secured in April 2024. A no-cost extension was recently received. As of April 2025, a total of 18 veterans with PD-MCI have completed the RCT phase. Data collection is ongoing and will be completed by March 2027. We expect the results of this study to be available by March 2028.

## Discussion

### Hypothesized Principal Findings

The overall research objective is to examine the therapeutic effects of rTMS on cognitive function in veterans with PD-MCI. The central hypothesis is that rTMS will safely improve cognitive function in PD-MCI. The first aim of the study is to determine the safety and feasibility of rTMS treatment. The hypothesis is that 10 high-frequency rTMS sessions will be (1) safe, as evidenced by the lack of difference in the number of research-related adverse events experienced by the active rTMS group compared to the sham rTMS group; and (2) feasible, as indicated by participant protocol completion rates. The second aim of the study is to determine the behavioral effects of rTMS. The hypothesis is that active rTMS, compared to sham rTMS, will enhance cognitive test performance after rTMS completion and at the 1-month follow-up.

### Comparison to Prior Work

Initial pilot RCTs found that multisession high frequency rTMS, targeted to the DLPFC improved depression (primary outcome) and cognitive function among patients with PD-N [14,15]. These promising results led to the design of this study protocol, which uses the 15-Hz parameter [15] but will assess cognitive function as the primary outcome among patients with PD-MCI.

Since the funding of this protocol, others have examined the effects of multisession rTMS on cognitive function (primary outcome) among patients with PD-MCI. Buard et al [16] reported no between-group differences on the total score on the Dementia Rating Scale–2 in a study that administered 20-Hz bilateral DLPFC rTMS at 90% the RMT for 10 sessions over 2 weeks. In contrast, 2 studies reported the effects of 10 sessions of high-frequency intermittent theta-burst (a patterned form of rTMS) targeted to the left DLPFC. Trung et al [17] reported improvement in visuospatial function after a 30-day follow-up in the active vs sham group. He et al [18] reported improvements in global cognitive function, immediate and delayed memory, and visuospatial ability. The improvements persisted for at least 3 months after the stimulation [18]. Finally, a meta-analysis of 14 studies suggests that multiple sessions of high-frequency rTMS over the DLPFC have positive effects on executive function in people living with PD [9]. Collectively, this evidence supports our selection of 10 sessions of high-frequency rTMS to improve cognitive function in PD-MCI.

### Strengths and Limitations

This is a rigorously designed RCT implementing strong blinding procedures. The placebo effect is a significant issue to consider in PD research in general and specifically in TMS [58,59] due to dopaminergic system involvement in expectation and reward processing. This may confound interpretation of TMS efficacy. To mitigate this, we use a double-blind, randomized sham-controlled design using a validated sham TMS coil that mimics the auditory and somatosensory experience of active stimulation without inducing cortical activity. Another strength of the study is that we apply gold-standard methods to define PD-MCI. Mood, such as depression and anxiety, can mediate test performance on cognitive assessments. Our study does not control for mood severity, which is a limitation of the study design; however, we will collect self-report mood assessments across time points. We will explore mood as a moderator of the cognitive effects of rTMS. Cautious interpretation of change in cognitive performance will have to be made in the context of mood.

### Dissemination Policy

Data will be presented and published in aggregate form and will be devoid of any individual identifiers. We plan to submit trial results to scientific peer-reviewed journals, and there are no publication restrictions. A CONSORT (Consolidated Standards of Reporting Trials) diagram will be included in the publication. We will follow the International Committee of Medical Journal Editors guidelines for authorship, and there is no intention of using professional writers. A veteran engagement panel is planned to involve patients in the reporting of the trial.

## Data Availability

The datasets generated or analyzed during this study will be available from the corresponding author on reasonable request.

## Appendix

Multimedia Appendix 1 Peer-review report by RRD8 Career Development Program - Panel I, VA Office of Rehabilitation Research Development and Translation (Department of Veterans Affairs, USA).

## Abbreviations

DLPFC: dorsolateral prefrontal cortex
DSMS: data safety monitoring sheet
FDA: Food and Drug Administration
IRB: institutional review board
MoCA: Montreal Cognitive Assessment
MRI: magnetic resonance imaging
NIH-EXAMINER: National Institutes of Health–sponsored Executive Abilities: Measures and Instruments for Neurobehavioral Evaluation and Research
PD: Parkinson disease
PD-MCI: mild cognitive impairment in Parkinson disease
PD-N: Parkinson disease with intact cognitive function
PI: principal investigator
RCT: randomized controlled trial
RMT: resting motor threshold
rTMS: repetitive transcranial magnetic stimulation
TMS: transcranial magnetic stimulation
VA: United States Department of Veterans Affairs
