# Supplementary material for: Repetitive Transcranial Magnetic Stimulation as a Cognitive Rehabilitation Approach for Veterans With Parkinson Disease and Mild Cognitive Impairment: Protocol for a Randomized Sham-Controlled Trial
Source: JMIR Res Protoc. 2026 Jun 29;15:e77241. doi: 10.2196/77241 (PMC13365885; doi:10.2196/77241)
Supplement: Multimedia Appendix 1 [file resprot_v15i1e77241_app1.pdf]

SUMMARY STATEMENT

PROGRAM CONTACT: **(Privileged Communication)** ***Release Date:*** **09/06/2018**
Revised Date:

Application Number: **1 IK2 RX002938-01A1**

Principal Investigator

KLETZEL, SANDRA LYNN

Applicant Organization: EDWARD HINES JR VA HOSPITAL

Review Group: **RRD8**
Career Development Program - Panel I

Meeting Date: **08/09/2018** ***RFA/PA:*** **RX18-016**
Council: **OCT 2018**
Requested Start: **11/01/2018**

Project Title: **rTMS as a Cognitive Rehabilitation Approach in Veterans with Parkinson's Disease**

SRG Action: **Impact Score:156**

Human Subjects: **30-Human subjects involved - Certified, no SRG concerns**
Animal Subjects: **10-No live vertebrate animals involved for competing appl.**
Gender: **1A-Both genders, scientifically acceptable**
Minority: **1A-Minorities and non-minorities, scientifically acceptable**
Children: **1A-Both Children and Adults, scientifically acceptable**
Clinical Research - not NIH-defined Phase III Trial

Project Year	Direct Costs Requested
1	176,388
2	178,616
3	180,888
4	183,206
5	185,570
TOTAL	904,668

ADMINISTRATIVE BUDGET NOTE: The budget shown is the requested budget and has not been adjusted to reflect any recommendations made by reviewers. If an award is planned, the costs will be calculated by VA Office of Research and Development (ORD) staff based on the recommendations outlined in the BUDGET COMMENT section and any relevant ORD service-specific limitations.

SUMMARY OF DISCUSSION:
A subcommittee of the Rehabilitation Research and Development Scientific Merit Review Board met in Plenary Session and reviewed the above proposal considering all internal and external reviews. This document summarizes the major points of the discussion concerning the proposed project. In any further development of this project, the investigator should consider carefully all the issues reflected in this Summary of Discussion as well as the more detailed comments in the individual critiques.

GENERAL COMMENTS:
The subcommittee agreed that Dr. Kletzel is an excellent CDA2 applicant who has the potential to become an independent VA rehabilitation scientist with unique bench to bedside skills. The reviewers appreciated Dr. Kletzel's responsiveness to the previous reviews and considered the application to be much improved in all areas. Dr. Kletzel's commitment to the VA and status as a co-Investigator on two clinical trials commendable. The subcommittee felt that the reconstitution of the mentoring team was appropriate and well-considered. Inclusion of specific plans for independence and future publications was praised, although the lack of specification of future proposal preparation in the applicant's planned activities/milestones was viewed as a weakness. The subcommittee expressed concern about the time commitment involved in the relatively considerable number of formal courses and workshops, especially considering the substantial increase in number of participants.

The planned research is highly relevant to Veterans and well-aligned with the training goals. The addition of post-hoc analyses to examine patient characteristics associated with responsiveness was an appropriate addition. However, the subcommittee felt that the responsiveness determination should be operationalized and taken into consideration relative to Aim 3. The subcommittee indicated that both responders and non-responders should be represented in the connectivity analyses; as currently planned, the rolling enrollment for Aim 3 would not guarantee representation of different degrees of responsiveness, or group (active-sham) equivalence. Overall, the subcommittee was enthusiastic about this applicant, the mentoring team, the training plan, and the proposed research.

SUGGESTIONS:
- Include specific plans and a timeline for future proposal submissions.
- Consider reducing the amount of didactic coursework and provide an analysis of time demands/allocation.
- More fully develop and operationalize the determination of responsiveness.
- Incorporate responsiveness into Aim 3.
- Reconcile inconsistencies in the training plan and research plan (e.g., duration of clinical rotation is described differently by the applicant versus the mentor; the number of participants in Aim 3 is indicated as both 12 per group and 6 per group).

COMMENTS ON THE BUDGET:
None.

DESCRIPTION (provided by applicant):
More than 100,000 US Veterans living with Parkinson's disease (PD) currently receive PD-related care and services from the VA. In addition to motor complications, PD is characterized by progressive cognitive decline leading to long-term functional impairment and diminished quality of life. Approximately 20-55% of PD patients will develop mild cognitive impairment (PD-MCI) while up to 80% will develop dementia (PD-D). Since PD-MCI is a risk factor for developing PD-D, there is a critical need to develop treatments to improve cognition and slow or stop progression to PD-D. Limited treatment options for PD-MCI and PD-D and the possible need to tailor treatments to Veterans makes this an important research topic. Repetitive transcranial magnetic stimulation (rTMS) shows promise as an effective cognitive neurorehabilitation treatment. To date, no rTMS studies have assessed the effect of rTMS on cognitive function in PD-MCI. Nor has there been PD neurophysiological studies using rTMS to examine neural plasticity in cognitive neural networks. This CDA2 seeks to fill this gap by

conducting a small scaled pilot randomized controlled trial (RCT) designed to assess the safety and therapeutic effects of rTMS on cognitive outcomes as well as on brain connectivity in Veterans with PD-MCI. PD-MCI participants will be randomized to either active rTMS or sham rTMS. Participants will complete a standardized neurocognitive battery assessment at baseline, endpoint (immediately after the 24-day intervention) and at a one-month follow-up. The primary outcome is change in executive function. Secondary outcomes include performance on other cognitive domain tasks and a proximal measure of real-life function that captures relevant functional changes related to cognitive impairment in PD. Multi-modal neuroimaging, in a subsample of participants, will be used to study neural connectivity changes induced by rTMS. Changes in resting state functional connectivity, grey matter volume via voxel-based morphometry and white matter integrity via diffusion tensor imaging will be assessed at baseline and endpoint. To inform how to optimize rTMS treatment in PD-MCI, these changes will be correlated with changes in cognitive performance. Dr. Kletzel is an excellent applicant for a CDA2. She has been building a strong foundation for establishing a career as a VA clinical neuroscientist in the field of neurorehabilitation. Her preclinical neuroscience background combined with her post-doctoral and CDA1 clinical research training makes her uniquely suited to successfully address the research objectives. Dr. Kletzel's long-term career goal is to develop and optimize cognitive rehabilitation treatments for Veterans with PD to improve their function and quality of life. Her immediate career goals are to conduct her first small clinical RCT and use these data both to contribute to the field of neurocognitive rehabilitation as well as to secure a VA Merit award as an independent investigator. To achieve these career goals, Dr. Kletzel has developed critical learning objectives set forth in her career plan. These include building upon and enhancing her clinical knowledge of PD and cognitive rehabilitation, expanding her skills of acquiring and analyzing multi-modal advanced neuroimaging data and developing her skills to conduct measurement research. Dr. Kletzel has assembled an impressive mentoring team to support her research and career plans. The team includes experts in the areas of PD and cognition, neuromodulation, neuroimaging and outcome measures. Completion of this CDA2 is expected to advance rehabilitative health care of Veterans who are affected by cognitive decline by providing necessary first steps towards developing a neuromodulatory cognitive intervention that has great potential to translate into clinical cognitive rehabilitation programs for Veterans with the goal of maximizing functional outcomes and improving quality of life.

PUBLIC HEALTH RELEVANCE:
The current CDA2 proposal addresses specific research interest areas to the VA including chronic diseases, psychiatric disorders, neurological dysfunction and rehabilitation outcomes. More than 100,000 US Veterans living with Parkinson's disease (PD) currently receive PD-related care and services from the VA. In addition to motor complications, PD is characterized by progressive cognitive decline leading to long-term functional impairment and disability. Dr. Kletzel's research potential, coupled with her commitment and passion for investigating cognitive function in Veterans with PD, makes this CDA2 application of great importance to Veteran health. Completion of this CDA2 is expected to advance rehabilitative health care of Veterans who are affected by cognitive decline by providing necessary first steps towards developing a neuromodulatory cognitive rehabilitation rTMS protocol that has great potential to translate into clinical cognitive rehabilitation programs for Veterans with the goal of maximizing functional outcomes.

CRITIQUE 1

Applicant:
Strengths:
- Excellent pre-clinical training in basic neuroscience, with experience with Parkinson's disease animal models.
- Promising neuroscientist, who will have a rare combination of bench to bedside skillset.
- Progress made in publications since last review – one manuscript with provisional acceptance, two manuscripts under review and a publication plan has been added to the proposal.

- Excellent letters of support.

Weaknesses: Number of publications remains limited, which is not surprising given the relatively short turn-around time from receipt of reviews and resubmission.

Mentor(s):
Strengths:
- Mentoring team has been reduced in size per recommendations: Drs. Pape, Bhaumik, and Bernard will remain as mentors. Dr. Rosenow (Director, Functional Neurosurgery at Northwestern and expert in PD) and Dr. Mallinson (psychometrician) have been added. Changes in mentoring team composition were carefully considered relative to Dr. Kletzel's needs/goals.
- Accomplished primary mentor who has a history of collaboration with the other members of the mentoring team.

Weaknesses: None noted.

Training Program:
Strengths:
- Training plan activities align well with overall career goal and with planned research.
- Much clearer delineation of activities that will lead to independence as a researcher.
- Specified plan for manuscript preparation is now included.
- Good balance in the training plan, including appropriate graduate courses, workshops, clinical rotations, grand rounds, hands-on training/experience in cognitive testing and rehabilitation, and neuromodulatory application (Dr. Pape's award), etc.

Weaknesses:
- A specific timeline for preparing a merit (or comparable) proposal submission is not included. There is only occasional passing mention of the CDA2 data serving as the basis of a merit submission. This reviewer is assuming that the TIGGR workshop will be a springboard for this preparation, but this is not clear.
- Meetings of the entire mentoring team are not described.

Scientific Merit:

Significance:
Strengths:
- Parkinson's disease (PD) is a relatively common neurological disease, affecting over 100,000 Veterans. Cognitive decline occurs in the majority of persons with PD. Unfortunately, there are limited pharmacological treatment options.
- rTMS may have potential for management of cognitive decline in PD.
- Findings may provide the necessary groundwork for later, more clinically impactful studies.

Weaknesses:
- The progression of cognitive decline in PD is not well-understood and consequently, it may be difficult to estimate the potential benefits of rTMS as a means for stemming progression in this short-term investigation. That is, unless there are cognitive gains in the treated group, interpretation of results may be difficult (e.g., lack of significant differences among the rTMS and sham group at four weeks post rTMS may not reflect possible longer-term benefits).
- Neuromodulation tends to have greater effects when used as an adjuvant/adjunct (to behavioral therapies) with other populations.

Approach:

Strengths:

- Comprehensive approach including safety, feasibility, cognitive, and brain-connectivity measures.
- Strong rationale supporting choice of target regions for stimulation.
- The “n” has been substantially increased (from 96 to 156) as a result of using a more conservative effect size and re-evaluating power analyses.
- The cognitive testing will now be completed across two testing days (two hours on one day and 80 minutes on a second day), which is an improvement (but, see below in weaknesses).
- Response concerning the prospect of negligible findings includes defining responsiveness relative to connectivity and conducting post-hoc analyses to examine patient characteristics associated with responsiveness (but, see below in weaknesses).

Weaknesses:

- Explanations concerning power calculations remain somewhat unclear (particularly for Aims 1 and 3).
- Aim 3 participants: 1) inconsistency concerning “n” - on page 29 - it is stated that there will be 12 participants per group and on page 33 it is stated that there will be six per group, and 2) procedures for selection of participants for Aim 3 (first 12 RCT participants who volunteer plus matching for next 12 willing participants) may result in insufficient representation of active or sham conditions (e.g., of the first 12 volunteers, there may be significantly more from one or the other group).
- Aim 1 – it is not clear what data will serve as the dependent measure(s) for feasibility. On page 33, there is an indication that responses to a questionnaire will be utilized as feasibility data, whereas on page 27, the feasibility outcome measure is stated to be “completion rates for rTMS sessions”, and feasibility is not addressed in the analyses. The data that will serve as the outcome measure for “safety” is somewhat unclear. It appears a safety questionnaire will be completed after each rTMS session, but how the data from the questionnaires will be managed is not indicated (number of items endorsed on the questionnaire? any consideration of severity of endorsed/observed items – is a seizure equivalent to a headache?).
- Geriatric Depression Scale (GDS) screening cut-off appears to be incorrect (e.g., “screens for depression, GDS<11”). Correct scoring interpretation is as follows: 0-4 = normal, 5-8 = mild, 9-11 = moderate, and 12-15 = severe.
- Determination of “full, partial, and non-responders” is not clear.
- Cognitive testing sessions are still somewhat lengthy. Although breaks will be offered, a more formal use of rest periods to combat cognitive fatigue would be preferable (e.g., enforced breaks of a specified length after a certain period of testing with beverages/refreshments provided).
- Did not address the possible outcome of no change in cognitive measures with both groups.

Resources:
Strengths: Supportive environment in terms of the scientific community, physical facilities, and clinical resources.

Weaknesses: None noted.

Protection of Human Subjects: Adequate, although suggest specifying break periods.

Inclusion of Women, Minorities, and Children: Exclusion of children scientifically justified. Very small “n” of women (n=10) is not discussed other than to indicate that this “n” reflects an increase from the previous submission and is due to small number of women Veterans with PD in previous studies. Doubtful that this small “n” is meaningful. Unsure how “n” for minorities was derived.

Critique of Vertebrate Animals Section:

	Yes	No

Research with vertebrate animals?		X

Biohazards and Radioisotopes: No comment.

Additional Review Criteria (unscored)

Budget (unscored)**:** Number of conferences has been reduced but still may exceed budget guidelines.

Data Management and Access Plan (for data sharing, unscored)**:** No concerns.

Overall Strengths:
- Applicant is a promising neuroscientist with excellent training.
- Applicant has an established relationship with and commitment to the VA.
- Strong mentoring team with collaborative history.
- Research plan is clinically relevant, innovative, and meshes well with VA "roll-out" of rTMS devices.
- Applicant has been responsive to previous reviews.

Overall Weaknesses:
- Concerns with publication productivity remain, with limited improvements since the previous submission. However, an explicit publication plan has now been included.
- Specific plans for proposal preparation and submission are not included.
- The possible outcome of no change in cognitive performance for both groups has not been adequately addressed.

CRITIQUE 2

Applicant: Dr. Kletzel is a strong CDA2 applicant, who has proven VA-centricity. She received her PhD (Loyola) in Neuroscience, with a focus on the effect of Parkinson's Disease (PD) medication on impulse decision making in a parkinsonian-like rodent model. After completing her doctoral training, her research transitioned to the clinical PD population. She received a competitive 2-year VA HSR&D funded postdoctoral fellowship at Hines VA. Upon completion of her postdoctoral fellowship, she received a VA RR&D CDA1 that focused on characterizing cognitive function in Veterans with PD and included prospective collection of resting state fMRI data in the PD population. The applicant has seven publications, four of which are first-author. In addition, she is Co-I on a DoD grant and a CDMRP grant that are related to neurorehabilitation after traumatic brain injury. Dr. Kletzel is also Chair of the American College of Rehabilitation Medicine (ACRM) Applied Cognition Geriatric Taskforce. The letters from the proposed mentors and others indicate strong support for Dr. Kletzel. In the previous submission of this CDA2, the limited number of publications was seen as a deficiency. Dr. Kletzel has made some progress to address this by receiving provisional acceptance of one additional manuscript and by submitting another two additional papers for review. In addition, she has modified her training plan to include manuscript milestones for her CDA2 to ensure continued productivity with publications.

Strengths:
- Dr. Kletzel has a very strong VA research background and her involvement as Co-I on two RCTs is impressive for an early-career investigator.
- Dr. Kletzel has an acceptable number of publications and has attempted to satisfy a deficiency noted during the previous review cycle by submitting three additional manuscripts for review, one of which has provisional acceptance.
- Dr. Kletzel has established expertise in preclinical Parkinsonian-like models and in clinical PD research, which is unique for an early-career investigator and a significant strength of the applicant.

Weaknesses: Continued improvement in the number of publications is necessary to develop into a successful independent investigator.

Mentor(s): The mentoring team has been revised appropriately and in accordance with suggestions received during the initial submission of the CDA2. The mentorship team is now comprised of five members who are senior scientists with expertise in rTMS and multi-site trials (Paper – Primary Mentor), PD clinical research (Rosenow – Physician/Scientist), Statistics (Bhaumik), Psychometrics (Mallinson), and Neuropsychology (Bernard).

Strengths:
- The applicant was responsive to initial reviews and revised the mentoring team accordingly.
- This is a very strong team, with extensive mentoring experience, funding, and publication histories.
- The role and need for each mentor is clearly justified and is re-iterated by each mentor in their letters of support.
- The letters from each mentor are very strong and indicate significant support for Dr. Kletzel as a CDA2 applicant.

Weaknesses:
- As indicated in the initial review of this application, it remains somewhat unclear why Dr. Kletzel requires another five years of postdoctoral training in the same laboratory where she has already served as a postdoc for four years.
- This concern is somewhat lessened from the initial submission because the Principal Investigator (PI) and the primary mentor have altered the mentoring plan and provided stronger justification for continued mentorship and training.
- However, nine years of postdoctoral training within the same laboratory appears somewhat excessive.

Training Program: The training plan is comprehensive and involves weekly or monthly meetings with mentors, along with the following training activities:
- Formal coursework:
  - Clinical Trials design (Loyola U).
  - Mechanisms Aging and Dementia (Northwestern).
  - Advanced Cognitive Neuroscience (UIC).
  - Cognitive Behavioral Assessment (UIC).
  - Biostatistics (UIC).
  - Psychometrics (online).
- Clinical exposure:
  - Shadowing – PD clinicians and Psychiatrists.
  - Movement Disorders Neuropsychiatry Rotation.
  - Clinical Practicum with Speech Pathology Service – Hines VA.
- Attendance at Seminars / Workshops:
  - Local seminars, Grand Rounds, Cyber Seminars.
  - 2-day ACRM Cognitive Rehabilitation workshop.
  - 3-day DTI, VBM, and rsFC analysis workshop.
  - 5-day DTI & rsFC analysis workshop.
  - rTMS for Rehab Workshop (MUSC).
  - VA TMS provider monthly phone calls.
  - Journal Club.
- Manuscript Milestones:
  - Publish two papers per year.
- Professional Development:
  - Manuscript Writing course (Madison, WI).
  - Writing group (Hines VA).

  - Mentoring/Research Development Advisory Group (Hines VA).
  - TIGRR workshop (MUSC).

Strengths:

- The training plan is comprehensive in that it addresses hands-on training in research techniques, didactic learning, and clinical training/observation.
- Improved rationale for all training components in included.
- Observation of PD patients during clinical visits is a strength of the training plan.
- The didactic training will improve Dr. Kletzel's knowledge in neurorehabilitation.

Weaknesses:

- As discussed in the initial review, the training plan appears burdensome in terms of the amount of time and work proposed. It is unclear if sufficient time exists for the applicant to successfully complete all aspects of training, while concurrently conducting the proposed research. A focus on performing research seems somewhat more important that additional didactic training, especially because of the limited publications that the applicant has at this stage in her career.
- Minor inconsistencies exist in the training plan. For example, the application states that Dr. Kletzel will participate in the 2-month (3 day/week) Neuropsychiatry in Movement Disorders rotation during three years of her CDA2 training. However, the letter from the Primary Mentor states this has been reduced to only one year of the CDA2 training.

Recommendations: Continue streamlining the training to include only the aspects of training that are most important for the development of Dr. Kletzel into an independent scientist, which will allow for additional hands-on research training.

Scientific Merit:

Significance: The significance of this work will be high if it is able to identify a positive effect from rTMS in the PD-MCI population. More than 100,000 US Veterans receive PD-related care from the VA, with no largely successful therapy available to improve cognitive function. Determining effective cognitive rehabilitation techniques has high clinical relevance. If successful, this research has the potential to limit the cognitive decline occurring in PD and/or improve cognitive recovery in those with PD-MCI.

Weaknesses:

- As noted in the original submission, the lack of preliminary data to demonstrate potential efficacy of rTMS on cognitive outcomes in (even in a few persons) with PD-MCI is a weakness.
- However, this has been somewhat alleviated given the vastly improved literature review indicating other groups have demonstrated rTMS efficacy in the general PD population in other pilot studies.

Approach: This is an extremely well-written proposal that was revised appropriately to address the previous reviews. The proposal will evaluate rTMS, a promising therapy for cognitive neurorehabilitation, in the PD-MCI population. No published work has evaluated whether rTMS improves cognition in persons with PD-MCI, although, other groups have reported some improvement in the general PD population in various pilot studies. Given these findings, assessing rTMS in PD-MCI appears to be a logical next step. The use of neuronavigation to locate specific target areas for rTMS is a strength of the proposal and should reduce variability in outcomes. The study design is significantly improved in this resubmission and most of the initial criticisms were adequately addressed.

Weaknesses:

- The rationale for assessing 110% rTMS appears sound. However, it is unclear what approach will be taken if 110% rTMS is shown to be unsafe or unfeasible in Aim 1.

- Aim 3 appears largely dependent upon the success of Aim 2. For example, if rTMS does not improve cognitive performance in PD-MCI, then it would be unlikely to observe changes in structural and functional connectivity via fMRI.
- The strategy for Aim 3 may prove problematic in terms of identifying structural and functional connectivity via fMRI if both high-responders and low-/non-responders are enrolled. How will variability in fMRI response be evaluated, given the relatively small sample proposed in Aim 3. Perhaps a better approach might be to directly compare fMRI responses between high-responders versus low-/non-responders to determine whether fMRI outcomes are associated with improved cognition in PD-MCI population.
- The feasibility of completing the proposed study is questionable, given that the power analysis indicates 156 participants (n=78/group) are required and that each participant will be required to complete one baseline data collection session, 10 rTMS sessions, an endpoint data collection session, and a follow-up data collection session (i.e., 14 total sessions). This effectively produces 2184 sessions (156 x 14 = 2184) over five years or 437 sessions per year.
- Minor inconsistencies remain in the application. For example, page 33 indicates n=6 active and n=6 sham rTMS will receive fMRI, while other parts of the application state n=12/group.

Resources: The environments at Hines VA and other associated medical centers and within the Pape Laboratory are considered excellent. All proposed resources are available.

Protection of Human Subjects: Appropriate.

Inclusion of Women, Minorities, and Children: Appropriate.

Critique of Vertebrate Animals Section: Not applicable.

	Yes	No
Research with vertebrate animals?		X

Biohazards and Radioisotopes: None.

Additional Review Criteria (unscored):

Budget (unscored)**:** The costs of training and travel seem somewhat excessive, and could be reduced if training components were lessened.

Data Management and Access Plan (for data sharing, unscored)**:** Appropriate.

Overall Strengths:
- Dr. Kletzel has strong qualifications, is well trained in PD research, has improving research productivity, and has strong VA-centricity.
- The mentoring team is a significant strength of the proposal and has strong qualifications.
- The training plan is comprehensive and has been streamlined to some degree, based on critiques received during the initial submission.
- The research environment and resources are excellent.
- The hypothesis that rTMS will improve cognitive function in PD-MCI appears logical and testable with the proposed study design.
- The rationale to pursue this research is improved with the revised literature review that is included in the resubmission.
- Overall, the proposal is well-designed, responsive to the previous critiques, and is designed appropriately to determine rTMS safety and efficacy in PD-MCI.
- If successful, it is likely that this proposal would result in future VA Merit or NIH submissions.

Overall Weaknesses:

- While the mentoring team has been appropriately changed, some additional justification is needed to explain why nine years of postdoctoral training in the same laboratory is necessary.
- Additionally, the feasibility of completing this study is questioned, given that >400 sessions per year are required within the 5-year time frame of this application.
- Feasibility is particularly worrisome, given that the didactic, hands-on training, shadowing, and workshops that are proposed represent a considerable time component that will take away from the time necessary to complete the proposed research.

CRITIQUE 3

Applicant: The PI demonstrates a clear commitment to the VA and its mission, as well as a clear potential to develop into an independent research scientist. Her long-term goal is to develop and optimize cognitive rehabilitation for Veterans with Parkinson's Disease (PD); her background in basic neuroscience research coupled with a more-recent transition into clinical work provides a strong foundation from which to carry out this type of work. She has been moderately productive with five first-author publications (a sixth has been accepted). A gap in publications is explained by the switch from basic research to clinical but a stronger publication history remains an important goal. The revised application describes an awareness of the limited publication history and presents a plan to significantly increase productivity including a strong commitment from each of the Mentors. For the most part, the letters of support (and recommendation) are very strong and indicate that the applicant has high potential to become an independent researcher at the VA. She is described as a 'motivated, enthusiastic and thoughtful early-career scientist committed to pursuing innovative and highly significant research questions.' She is often referred to as having excellent organizational skills and good attention to detail. She has performed service to the research community and currently serves as chair of a Measurement Networking Group.

Mentor(s): A strong and comprehensive group of mentors have been assembled; they provide expertise that is well aligned with the applicant's CDA2 goals. Many of the mentors are already part of an existing project group focused on Aims similar to that of this CDA2. The applicant's existing involvement in that group has allowed her to develop relationships with the individual mentors and there is clear evidence of much interaction already, particularly as it relates to shaping the proposed Aims, Methods and Training Plan. The Primary Mentor was previously a CDA1 and CDA2 and has successfully mentored numerous trainees previously. Individual mentors have clearly delineated roles in the applicant's training and the overall group nicely covers the range of expertise needed to support the proposed efforts. Despite Dr. Rosenow's strong credentials, it was harder to discern how his specific expertise aligned with the goals of Aim 1 and whether he had previously performed safety and feasibility studies with rTMS. Also, he has mentored fewer trainees than the other mentors and most of them have been medical students or fellows. Nevertheless, the scope of the project is similar enough to ongoing efforts from the larger project team, alleviating concerns that this problem would hinder progress.

Training Program: The revised training program is excellent. It provides a clear description of the comprehensive plan for training. Training for the specific skill sets needed for success are clearly delineated and align well with the proposed research and other goals of the applicant. The plan is strongly supported by the letters from individual Mentors with each elucidating their specific roles and commitments to the applicant. The overall presentation suggests the Training Program has been carefully prepared and involved coordination from all team members. The plan includes many didactic courses and/or training programs, regular interactions with all mentors and appropriate oversight from key mentors; there is some small concern that the overall training plan is overly ambitious. The overall Training Program provides a high level of confidence that the mentors are committed to the continued

success of the applicant. Finally, the applicant's research interests are like those of many of the mentors but they do not directly overlap; this is ideal in that it optimizes training while still providing a path to independence for the applicant.

Scientific Merit:

Significance: Many people with Parkinson's Disease experience cognitive decline and/or dementia over the course of their disease. Medications used to address cognitive impairment in PD have not been effective. The focus of the CDA2 research Aims is on the development of an alternative intervention for these patients. Specifically, the use of repetitive transcranial magnetic stimulation (rTMS) will be explored for its neuromodulatory effect. RTMS is a well-established treatment modality for many other neurological diseases and the primary mentor (and others) have been using it for the treatment of some of these. The investigation of rTMS for PD is a natural extension of these previous efforts and is ideal in that the applicant's efforts can be supported by the ongoing use of this modality while still leaving her with a path to independence. Development of a non-invasive, non-pharmacological treatment for cognitive decline in PD patients would be of considerable benefit to Veterans and the general population as well.

Approach: The approach is solid. For the most part, the aims are well-developed and clearly described. The aims cover safety and feasibility, behavioral responses and then exploration of the anatomical changes induced by the rTMS treatment. This comprehensive approach is attractive in that it builds on the expertise of the applicant in both basic and clinical research while simultaneously allowing her to continue developing expertise in running a RCT. There was some previous concern about the potential for equivocal effectiveness, e.g. whether control and treatment groups might present similar results; it was still not entirely clear as to how this would be addressed.

Resources: No concerns.

Protection of Human Subjects: No concerns. Mentors have strong expertise in performing these types of studies which further alleviates any concern.

Inclusion of Women, Minorities, and Children: Not applicable.

Critique of Vertebrate Animals Section:

	Yes	No
Research with vertebrate animals?		x

Biohazards and Radioisotopes: Not applicable.

Additional Review Criteria (unscored)

Budget (unscored)**:** No concerns.

Data Management and Access Plan (for data sharing, unscored)**:** No concerns.

Overall Strengths:
- Strong applicant, strong mentors and a very strong training plan.
- The research focus addresses an important problem in Veterans (and the general population) and the research is in an area of growing interest within the VA.

Overall Weaknesses: Minor concerns about one of the Mentors but these are mitigated by the strength of the rest of the team and their consistent productivity over many years.

MEETING ROSTER

Career Development Program - Panel I
Rehabilitation Research and Development Parent IRG
Office of Research & Development
RRD8
08/09/2018

CHAIRPERSON(S)

WECHT, JILL M., EDD
RESEARCH ASSOCIATE/PRINCIPAL INVESTIGATOR
SPINAL CORD DAMAGE RESEARCH CENTER
JAMES J. PETERS VA MEDICAL CENTER (BRONX)
PROFESSOR OF MEDICINE AND REHABILITATION MEDICINE
MOUNT SINAI SCHOOL OF MEDICINE
BRONX, NY 10468

MEMBERS

BRUNS, TIMOTHY M., PHD *
ASSISTANT PROFESSOR
DEPT OF BIOMEDICAL ENGINEERING
UNIVERSITY OF MICHIGAN
ANN ARBOR, MI 48109

CARDOZO, CHRISTOPHER, MD, PHD
STAFF PHYSICIAN
BRONX VA MEDICAL CENTER
CENTER OF EXCELLENCE FOR THE
MEDICAL CONSEQUENCES OF SCI
BRONX, NY 10468

FRIED, SHELLEY, PHD *
RESEARCH SCIENTIST
BOSTON VA MEDICAL CENTER
INSTRUCTOR IN NEUROSCIENCE
DEPARTMENT OF NEUROBIOLOGY
MASSACHUSETTS GENERAL HOSPITAL
BOSTON, MA 02114

GUSTAFSON, KENNETH J., PHD *
ASSOCIATE DIRECTOR
CLEVELAND VA MEDICAL CENTER
CLEVELAND FES CENTER
ASSOC PROF, DEPT OF BIOMEDICAL ENG & UROLOGY
CASE WESTERN RESERVE UNIVERSITY
CLEVELAND, OH 44106

HOLT, AVRIL GENENE, PHD *
HEALTH SCIENCE SPECIALIST
DETROIT VA MEDICAL CENTER
ASSOCIATE PROFESSOR
DEPARTMENT OF ANATOMY AND CELL BIOLOGY
WAYNE STATE UNIVERSITY
DETROIT, MI 48201

JAN, YIH-KUEN, PHD *
ASSOCIATE PROFESSOR
COLLEGE OF APPLIED HEALTH SCIENCES
UNIVERSITY OF ILLINOIS AT URBANA-CHAMPAIGN
CHAMPAIGN, IL 61820

MITCHELL, BRAXTON D. JR, PHD *
PROFESSOR
DEPARTMENT OF MEDICINE
EPIDEMIOLOGY AND PUBLIC HEALTH
UNIVERSITY OF MARYLAND
BALTIMORE, MD 21201

OBENAUS, ANDRE, PHD
PROFESSOR
DEPARTMENT OF PEDIATRICS
UNIVERSITY OF CALIFORNIA, IRVINE
IRVINE, CA 92697

REX, TONIA S, PHD *
ASSOCIATE PROFESSOR
VANDERBILT BRAIN INSTITUTE
VANDERBILT UNIVERSITY
NASHVILLE, TN 37232

SWARTZWELDER, H SCOTT, PHD *
PROFESSOR
DEPT OF PSYCHIATRY AND BEHAVIORAL SCIENCES
DUKE UNIVERSITY MEDICAL CENTER
DURHAM, NC 27710

WAMBAUGH, JULIE L., PHD *
RESEARCH CAREER SCIENTIST
SALT LAKE CITY VA MEDICAL CENTER
PROFESSOR
COMMUNICATION SCIENCES AND DISORDERS
UNIVERSITY OF UTAH
SALT LAKE CITY, UT 84112

WILLETT, NICK J, PHD *
BIOMEDICAL ENGINEER
ATLANTA VAMC
ASSISTANT PROFESSOR
DEPARTMENT OF ORTHOPAEDICS
SCHOOL OF MEDICINE AT EMORY UNIVERSITY
DECATUR, GA 30033

YAMAGUCHI, DEAN T, MD, PHD
ASSOCIATE CHIEF OF STAFF, RESEARCH AND
DEVELOPEMENT
GREATER LOS ANGELES VA HEALTHCARE SYSTEM
PROFESSOR OF MEDICINE
UNIVERSITY OF CALIFORNIA, LOS ANGELES
LOS ANGELES, CA 90073

YARROW, JOSHUA F., PHD *
RESEARCH HEALTH SCIENTIST
MALCOLM RANDALL VA MEDICAL CENTER
ASSISTANT SCIENTIST
DEPT OF APPLIED PHYSIOLOGY AND KINESIOLOGY
UNIVERSITY OF FLORIDA
GAINESVILLE, FL 32608

EXECUTIVE SECRETARY

BOUWER, H. G. ARCHIE, PHD
DEPUTY CHIEF OF STAFF
PORTLAND VA HEALTH CARE SYSTEM
SOM-MOLECULAR MICROBIOLOGY & IMMUNOLOGY DEPT
OREGON HEALTH SCIENCES UNIVERSITY
PORTLAND, OR 97213

SCIENTIFIC REVIEW OFFICER

GROER, SHIRLEY, PHD
SCIENTIFIC PROGRAM MANAGER
DEPARTMENT OF VETERANS AFFAIRS
OFFICE OF RESEARCH AND DEVELOPMENT
REHABILITATION RESEARCH AND DEVELOPMENT SERVICE
WASHINGTON, DC 20420

* Temporary Member. For grant applications, temporary members may participate in the entire meeting or may review only selected applications as needed.

Consultants are required to absent themselves from the room during the review of any application if their presence would constitute or appear to constitute a conflict of interest.
